# Application of ensemble clustering and survival tree analysis for identifying prognostic clinicogenomic features in patients with colorectal cancer from the 100,000 Genomes Project

**DOI:** 10.1186/s13104-021-05789-0

**Published:** 2021-10-02

**Authors:** Yuguo Wei, Nikolaos Papachristou, Stefanie Mueller, Genomics England Research Consortium, J. C. Ambrose, P. Arumugam, M. Bleda, F. Boardman-Pretty, C. R. Boustred, H. Brittain, M. J. Caulfield, G. C. Chan, T. Fowler, A. Giess, A. Hamblin, S. Henderson, T. J. P. Hubbard, R. Jackson, L. J. Jones, D. Kasperaviciute, M. Kayikci, A. Kousathanas, L. Lahnstein, S. E. A. Leigh, I. U. S. Leong, F. J. Lopez, F. Maleady-Crowe, L. Moutsianas, M. Mueller, N. Murugaesu, A. C. Need, P. O’Donovan, C. A. Odhams, C. Patch, D. Perez-Gil, M. B. Pereira, J. Pullinger, T. Rahim, A. Rendon, T. Rogers, K. Savage, K. Sawant, R. H. Scott, A. Siddiq, A. Sieghart, S. C. Smith, A. Sosinsky, A. Stuckey, M. Tanguy, E. R. A. Thomas, S. R. Thompson, A. Tucci, E. Walsh, M. J. Welland, E. Williams, K. Witkowska, S. M. Wood, Wai Hoong Chang, Alvina G. Lai

**Affiliations:** 1 Institute of Health Informatics, University College London, London, UK; 2 Genomics England, London, UK

## Abstract

**Objective:**

The objective of this study was to employ ensemble clustering and tree-based risk model approaches to identify interactions between clinicogenomic features for colorectal cancer using the 100,000 Genomes Project.

**Results:**

Among the 2211 patients with colorectal cancer (mean age of diagnosis: 67.7; 59.7% male), 16.3%, 36.3%, 39.0% and 8.4% had stage 1, 2, 3 and 4 cancers, respectively. Almost every patient had surgery (99.7%), 47.4% had chemotherapy, 7.6% had radiotherapy and 1.4% had immunotherapy. On average, tumour mutational burden (TMB) was 18 mutations/Mb and 34.4%, 31.3% and 25.7% of patients had structural or copy number mutations in *KRAS*, *BRAF* and *NRAS*, respectively. In the fully adjusted Cox model, patients with advanced cancer [stage 3 hazard ratio (HR)  =  3.2; p  <  0.001; stage 4 HR  =  10.2; p  <  0.001] and those who had immunotherapy (HR  =  1.8; p  <  0.04) or radiotherapy (HR  =  1.5; p  <  0.02) treatment had a higher risk of dying. The ensemble clustering approach generated four distinct clusters where patients in cluster 2 had the best survival outcomes (1-year: 98.7%; 2-year: 96.7%; 3-year: 93.0%) while patients in cluster 3 (1-year: 87.9; 2-year: 70.0%; 3-year: 53.1%) had the worst outcomes. Kaplan–Meier analysis and log rank test revealed that the clusters were separated into distinct prognostic groups (p  <  0.0001). Survival tree or recursive partitioning analyses were performed to further explore risk groups within each cluster. Among patients in cluster 2, for example, interactions between cancer stage, grade, radiotherapy, TMB, *BRAF* mutation status were identified. Patients with stage 4 cancer and TMB  ≥  1.6 mutations/Mb had 4 times higher risk of dying relative to the baseline hazard in that cluster.

**Supplementary Information:**

The online version contains supplementary material available at 10.1186/s13104-021-05789-0.

## Introduction

An evaluation of cancer drug approvals by the European Medicines Agency (EMA) found that 57% of drugs entered the market with limited evidence of survival benefits where at 3 years after market entry, survival gains in patients receiving 33 of the 39 cancer drugs were marginal [1]. Long-term follow-up analyses of data from trials in the post-market entry period are rare. Moreover, only 26% of randomised controlled trials (RCTs) investigated extension of life as the primary outcome [1] despite the EMA’s recommendation that overall survival is the most crucial outcome for investigating efficacy and safety of oncology drugs [2].

In light of these issues, population-based health records may help advance the long-term post-market evaluation of cancer drugs and support the identification and approval of new indications. Since the inception of the 21st Century Cures Act that aims to accelerate the development and innovation of medicines, the Food and Drug Administration created a framework for evaluating the use of real-world data (RWD) to support drug trials [3]. Overall survival in patients with cancer is well studied, particularly in the context of how genetic and transcriptomic alterations in tumours affect patient outcomes. Harnessing data from the International Cancer Genome Consortium and The Cancer Genome Atlas, there have been concerted efforts by the cancer community to develop genetic signatures that are predictive of overall survival rates [4–10]. Linking genetic data with electronic health records (EHRs) may allow further exploration of associations between clinical characteristics and tumour genetic profiles.

We seek to evaluate whether a clinicogenomic dataset from Genomics England could be useful for predicting survival outcomes based on clinical and genetic features in patients with colorectal cancer. We employed an ensemble clustering approach and survival tree analysis to identify clinicogenomic features that are indicative of prognosis.

## Main text

### Materials and methods

#### Dataset

Clinical and genome sequence data from 2211 patients with colorectal cancer was used.

##### Clinical data

Clinical data consisted of data from secondary care, the National Cancer Registration and Analysis Service and the Office for National Statistics (ONS). We obtained patient demographic details, age at cancer diagnosis, sex, tumour grade, tumour, nodes metastasis (TNM) stage and information on cancer therapy. Cancer type was defined based on site and morphology of cancer coded in ICD-O2 and ICD-10. Tumour grade was defined as G1 (well differentiated), G2 (moderately differentiated) and G3 (poorly differentiated). Cancer treatment was categorised as surgery, chemotherapy, radiotherapy and immunotherapy. Mortality data were obtained from the ONS registry.

##### Genetic data

Tumour mutational burden (TMB) was computed as the number of somatic non-synonymous small variants per megabase (Mb) of coding sequence. Only variants meeting the quality threshold criteria were included in TMB ascertainment (detailed calculations of variant quality metrics have been described previously) [11]. Somatic structural variants (SVs) and copy number variants (CNVs) for *BRAF, KRAS* and *NRAS* were obtained using the R package ‘getSVCNVperGene’. SVs and long indel (>  50 bp) calling were performed using the Manta Structural Variant Caller [12]. CNVs were called using the Canvas algorithm that identified genomic regions that had been lost or gained and investigated minor allele frequencies and coverage to determine copy number [13].

#### Ensemble clustering and validation

To perform clustering on mixed data types (i.e., numerical and categorical data), we first analysed dissimilarity between observations using the Gower distance. We employed four clustering algorithms: partitioning around medoids (PAM), hierarchical clustering (i.e., divisive analysis or DIANA), Fuzzy C-means (FCM) and k-means. We employed ensemble clustering (consensus clustering) [14] to merge results from multiple clustering algorithms above using the diceR package. The ‘dice’ function was used to perform consensus clustering across subsamples and algorithms for a different number of clusters (k). The number of subsamples was specified as five and the consensus function to use was specified as the cluster-based similarity partitioning algorithm. Internal cluster validation indices were used to assess the performance by considering the separability and compactness of the clusters. We selected the C-index, silhouette coefficient, compactness and connectivity indices for validation. The relative ranks of each algorithm across the internal indices were considered and their sum was computed. Algorithms below 75% for the sum rank were trimmed. Post trimming, algorithms were reweighted based on their internal index magnitudes and fed into the consensus function.

#### Survival analyses

Prior to clustering, we applied the Cox proportional hazards regression analysis to estimate overall survival outcomes for each clinicogenomic feature. All hazard ratios were fully adjusted for all other features investigated. To examine potential interactions between clinicogenomic predictors, we performed survival tree (recursive partitioning) analyses [15] for each cluster using the rpart package. Tree-based models allowed the visualisation of decision rules for predicting an outcome for different patient groups within each cluster. Survival data were pre-scaled to fit an exponential model in that the predicted risk in the root node is fixed to 1.0. Relative risk estimates in other nodes were reported as relative to the survival in the root node (i.e., relative to the baseline hazard). Mean deviance was ascertained to measure the variability among all observations that reached a specified node.

## Results

### Clinicogenomic features of the colorectal cancer cohort

**Fig. 1:**
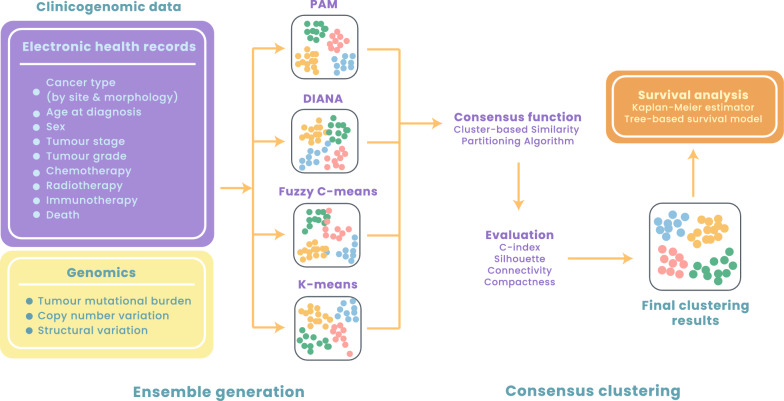
Schematic diagram depicting the study design

The Genomics England cohort consisted of 2211 patients diagnosed with colorectal cancer (see Fig. 1 for the study design). Of these patients, 59.7% were men and the mean age of diagnosis was 67.7 (Additional file 1). The proportions of patients with colorectal cancer diagnosed at different stages were as follow: stage 1 (16.3%), stage 2 (36.3%), stage 3 (39.0%) and stage 4 (8.4%). Patients were also classified according to tumour: grade 1 (3.0%), grade 2 (81.9%) and grade 3 (15.1%). Almost all patients underwent surgical intervention (99.7%), while 47.4% of patients underwent chemotherapy, 7.6% had radiotherapy and 1.4% had immunotherapy. With regards to genetic features, we explored tumour mutational burden (TMB) and observed that patients had 18 mutations/Mb on average (Additional file 1). We also explored structural variants and copy number variants and found that 34.4%, 31.3% and 25.7% of patients had mutations in *KRAS*, *BRAF* and *NRAS* respectively (Additional file 1). We performed Cox regression analyses where all hazard ratios (HRs) were fully adjusted for all other features considered. In the fully adjusted model, when considering cancer stage (with stage 1 as the reference), patients who were at stage 3 (HR  =  3.2; p  <  0.001) and stage 4 (HR  =  10.2; p  <  0.001) had a significantly higher risk of death, while stage 2 patients did not show any increase in risk (HR  =  1.3; p  =  0.29). Additionally, immunotherapy (HR  =  1.8; p  <  0.04) and radiotherapy (HR  =  1.5; p  <  0.02) were significantly associated with poorer survival outcomes (Additional file 2).

### Ensemble clustering and validation

Employing resampling of the four clustering algorithms (K-means, PAM, DIANA and fuzzy C-means) on five replicates of the dataset, the optimal number of clusters was found to be between four and six. Internal cluster validity indices (i.e., C-index, silhouette coefficient, compactness and connectivity) for cluster validation were assessed to compare results from the varying number of clusters (k) (Additional file 3). Based on the cluster validity indices, k  =  4 was found to be optimal based on compactness and connectivity (Additional file 4). We employed the ensemble clustering approach to consider the relative ranks of each of the four clustering algorithms across all internal validity indices to compute the overall rank sum to generate a final ensemble with the largest silhouette coefficient and lowest connectivity (Additional file 4).

### Descriptive features of the clusters and overall survival outcomes

**Fig. 2:**
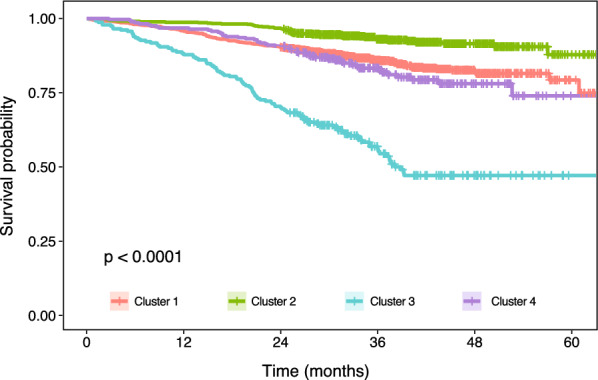
Overall survival outcomes in patients with colorectal cancer stratified using the ensemble clustering approach

Descriptive statistics of the clusters were provided in Additional file 5. Cluster 1 featured patients with high TMB (mean  =  32.3 non-synonymous somatic mutations per Mb). The proportion of patients having stage 1 or stage 2 tumours were as follow: cluster 1 (69.2%), cluster 2 (32.2%), cluster 3 (8.7%) and cluster 4 (80.9%) (Additional file 5). Cluster 2 consisted of the highest proportion of patients who had chemotherapy (75.2%) while cluster 4 had the least number of patients who had chemotherapy (27.7%) (Additional file 5). Cluster 4 had the highest proportion of patients with mutations in *KRAS* (60.8%), *BRAF* (55.8%) and *NRAS* (54.7%). We estimated overall survival rates at 1 year, 2 years and 3 years post-diagnosis for each cluster. Patients in cluster 2 had the best survival outcomes (1-year: 98.7%; 2-year: 96.7%; 3-year: 93.0%) while patients in cluster 3 (1-year: 87.9; 2-year: 70.0%; 3-year: 53.1%) had the worst outcomes (Additional file 5). We generated a Kaplan–Meier plot and to illustrate the overall survival outcomes stratified by the clusters generated from ensemble clustering. Log rank test revealed that the clusters were separated into distinct prognostic groups (p  <  0.0001) (Fig. 2).

### Survival tree analyses within each prognostic cluster offered an additional resolution to stratify patients into risk group by clinicogenomic features

**Fig. 3:**
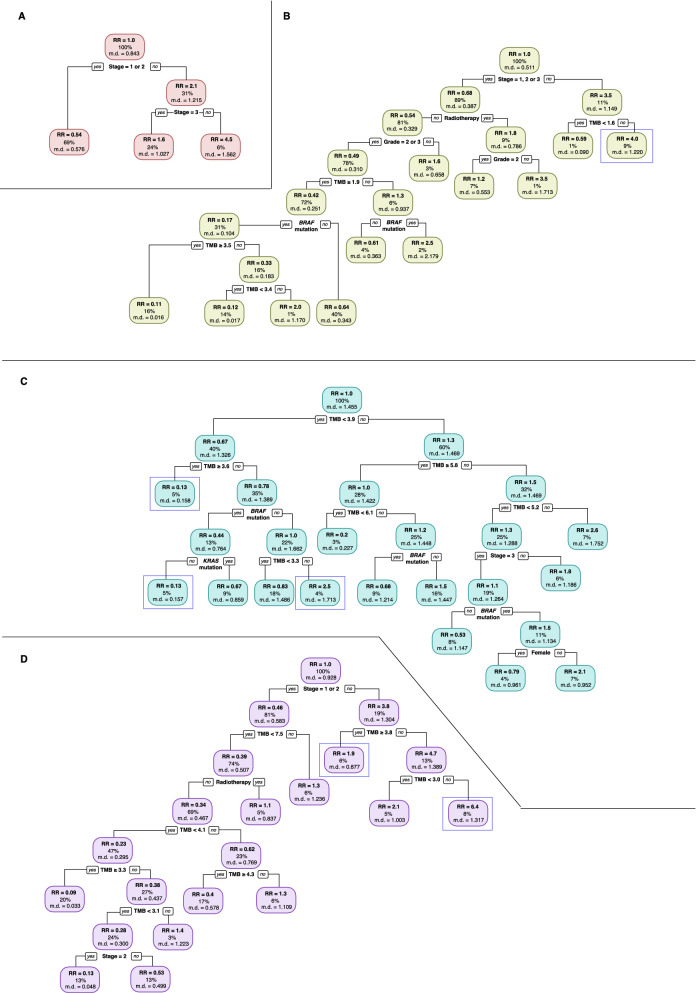
Survival tree analyses for each of the four prognostic clusters. **A** Cluster 1, **B** cluster 2, **C** cluster 3 and **D** cluster 4. Proportion of patients within each node are indicated as a percentage. Relative risk (RR) for each node is indicated, along with the mean deviance (md) value. Nodes described in the results section are highlighted in blue boxes

We performed survival tree (recursive partitioning) analyses to garner additional insights into the potential interactions between clinicogenomic predictors. Survival trees allowed the identification of interactions between clinicogenomic predictors by grouping subjects according to their survival profiles. Survival trees for each cluster were shown in Fig. 3. For cluster 1, cancer stage was found to be a significant predictor of overall survival (Fig. 3A). For cluster 2, cancer stage, grade, radiotherapy, TMB, *BRAF* mutation status were important predictors (Fig. 3B). Survival tree for cluster 3 featured TMB, *BRAF* and *KRAS* mutation status and cancer stage as predictors (Fig. 3C). For cluster 4, stage, radiotherapy and TMB were found to be important (Fig. 3D).

Relative risk estimates were shown in the survival trees for each node as relative to the survival in the root node. In the survival tree for cluster 2, the rightmost terminal node had a relative death rate of 4 times the overall rate and was defined by stage 4 and TMB  ≥  1.6 (Fig. 3B). For cluster 3, TMB was found to be the most important predictor of overall survival with a cut-off value of 3.9 mutations/Mb. Patients with TMB  <  3.9 mutations/Mb were further split by TMB and *BRAF* mutation status, in which the group with TMB  <  3.6 mutations/Mb, no BRAF mutation and TMB  ≥  3.3 mutations/Mb had a relative death rate of 2.5 times the overall rate (Fig. 3C). In contrast, we observed a low relative risk for death of 0.13 in patients with TMB  <  3.9 but TMB  ≥  3.6 mutations/Mb (Fig. 3C). Similarly, a relative risk of 0.13 is also observed in another group of patients having TMB  <  3.9 and also TMB  <  3.6 mutations/Mb, followed by having BRAF mutation and no KRAS mutation (Fig. 3C). Survival tree for cluster 4 revealed that patients were initially split by cancer stage, where the left side of the tree consisted of patients at stage 1 or 2, while the right side was for patients at stage 3 or 4 (Fig. 3D). Then, patients at stage 3 or 4 were split by TMB with a cut-off value of 3.8 mutations/Mb. Patients with TMB  ≥  3.8 had a relative death rate of 1.9 times. Patients with TMB  <  3.8 were further split by TMB with a cut-off of 3.0, and patients with TMB  ≥  3.0 but  <  3.8 had a relative death rate of 6.4 times the overall rate (Fig. 3D).

## Discussion

We demonstrated the feasibility of predicting overall survival outcomes in patients with colorectal cancer using ensemble clustering and survival tree analyses on a clinicogenomic dataset. Large-scale national estimates of colorectal cancer survival rates have mostly focused on cancer survival by stage using staging information collected from population-based cancer registries [16]. Independently, other studies have investigated the role of genomic biomarkers on cancer survival outcomes [17–23]. Our study demonstrated that clinicogenomic features can be employed to provide additional resolution for stratifying patients into risk groups not currently afforded by staging information alone. We have identified four prognostic clusters using ensemble clustering. As each cluster consists of a heterogeneous group of patients, subsequent survival tree analyses within the clusters revealed the different contributions of cancer stage/grade, radiotherapy treatment, TMB, *BRAF* or *KRAS* mutation status in predicting the relative risk of death in patients with colorectal cancer.

## Limitations

First, the study is underpowered to investigate prognosis in relation to specific cancer therapy. Second, we have only investigated mutation profiles of *KRAS*, *BRAF* and *NRAS* as an initial proof of concept. Third, analyses were performed only in patients with complete staging information. Future work may explore imputation methods to address missing data. Fourth, the selection of patients for recruitment into the 100,000 Genomes Project may introduce a selection bias for individuals with access to specific healthcare services.

## Supplementary Information

**Additional file 1: **Baseline characteristics of patients with colorectal cancer in the 100,000 Genomes Project cohort.

**Additional file 2: **Hazard ratios and 95% confidence intervals for overall survival.

**Additional file 3: **Internal cluster validity indices for k = 5 and 6.

**Additional file 4: **Internal cluster validity indices for k = 4.

**Additional file 5: **Clinicogenomic features after ensemble clustering.

## Data Availability

The datasets supporting the conclusions of this article are included within the article and its additional files. Access to the 100,000 Genomes Project database (https://www.genomicsengland.co.uk/) is subjected to approval.

## Abbreviations

RWD: Real-world data
RCT: Randomised control trial
EMA: European Medicines Agency
HER: Electronic health record
TMB: Tumour mutational burden
HR: Hazard ratio
TNM: Tumour, nodes, metastasis
SV: Structural variant
CNV: Copy number variant
PAM: Partition around medoids
DIANA: Divisive analysis
FCM: Fuzzy C-means
