# Supplementary material for: Application of ensemble clustering and survival tree analysis for identifying prognostic clinicogenomic features in patients with colorectal cancer from the 100,000 Genomes Project
Source: BMC Res Notes. 2021 Oct 2;14:385. doi: 10.1186/s13104-021-05789-0 (PMC8487486; doi:10.1186/s13104-021-05789-0)
Supplement: Supplementary file 1 — Additional file 1: Baseline characteristics of patients with colorectal cancer in the 100,000 Genomes Project cohort. [file 13104_2021_5789_MOESM1_ESM.pdf]

Additional file 1: Baseline characteristics of patients with colorectal cancer

Features	Alive (n = 1856)		Dead (n = 355)		Total (n = 2211)	
**Male (%)**	1101	59.3%	218	61.4%	1319	59.7%
**Age groups (%)**						
≤ 30	4	0.2%	0	0.0%	4	0.2%
31-50	155	8.4%	22	6.2%	177	8.0%
51-70	879	47.4%	162	45.6%	1041	47.1%
≥ 71	818	44.1%	171	48.2%	989	44.7%
**Tumour stage (%)**						
1	339	18.3%	22	6.2%	361	16.3%
2	729	39.3%	73	20.6%	802	36.3%
3	692	37.3%	170	47.9%	862	39.0%
4	96	5.2%	90	25.4%	186	8.4%
**Tumour grade (%)**						
1	57	3.1%	10	2.8%	67	3.0%
2	1548	83.4%	262	73.8%	1810	81.9%
3	251	13.5%	83	23.4%	334	15.1%
**Treatment (%)**						
Surgery	1852	99.8%	352	99.2%	2204	99.7%
Chemotherapy	830	44.7%	217	61.1%	1047	47.4%
Radiotherapy	124	6.7%	45	12.7%	169	7.6%
Immunotherapy	16	0.9%	14	3.9%	30	1.4%
**Tumour mutational burden (mean, SD)**	17.7	±35.0	19.4	±40.9	18.0	±35.9
***KRAS* mutation (%)**	651	35.1%	110	31.0%	761	34.4%
***BRAF* mutation (%)**	590	31.8%	103	29.0%	693	31.3%
***NRAS* mutation (%)**	484	26.1%	85	23.9%	569	25.7%
