# Supplementary material for: Application of ensemble clustering and survival tree analysis for identifying prognostic clinicogenomic features in patients with colorectal cancer from the 100,000 Genomes Project
Source: BMC Res Notes. 2021 Oct 2;14:385. doi: 10.1186/s13104-021-05789-0 (PMC8487486; doi:10.1186/s13104-021-05789-0)
Supplement: Supplementary file 2 — Additional file 2: Hazard ratios and 95% confidence intervals for overall survival. [file 13104_2021_5789_MOESM2_ESM.pdf]

Additional file 2: Hazard ratios and 95% confidence intervals for overall survival

Features	Fully adjusted hazard ratio	Lower CI	Upper CI	P value
**Female**	0.89	0.72	1.11	0.31
**Age**	1.03	1.02	1.05	< 0.001
**Stage 1 (reference)**				
**Stage 2**	1.30	0.80	2.10	0.29
**Stage 3**	3.20	1.99	5.15	**< 0.001**
**Stage 4**	10.20	6.16	16.90	**< 0.001**
**Grade 1 (reference)**				
**Grade 2**	1.03	0.55	1.96	0.92
**Grade 3**	1.67	0.85	3.26	0.13
**Chemotherapy**	1.03	0.79	1.35	0.81
**Immunotherapy**	1.77	1.02	3.08	**0.04**
**Radiotherapy**	1.46	1.06	2.01	**0.02**
**Tumour mutational burden**	1.00	1.00	1.01	0.11
**KRAS mutation**	1.15	0.91	1.45	0.23
**BRAF mutation**	1.01	0.79	1.28	0.94
**NRAS mutation**	1.09	0.85	1.40	0.47
