# Supplementary material for: Application of ensemble clustering and survival tree analysis for identifying prognostic clinicogenomic features in patients with colorectal cancer from the 100,000 Genomes Project
Source: BMC Res Notes. 2021 Oct 2;14:385. doi: 10.1186/s13104-021-05789-0 (PMC8487486; doi:10.1186/s13104-021-05789-0)
Supplement: Supplementary file 3 — Additional file 3: Internal cluster validity indices for k = 5 and 6. [file 13104_2021_5789_MOESM3_ESM.pdf]

Additional file 3: Internal cluster validity indices for k = 5 and 6

Algorithm	C-index	Silhouette	Compactness	Connectivity
			**k = 5**	
**PAM**	0.294	0.064	2.212	1064.2
**DIANA**	0.356	0.103	2.334	744.8
**Fuzzy c-means**	0.147	0.150	1.909	495.2
**K-means**	0.163	0.086	1.995	804.0
			**k = 6**	
**PAM**	0.274	0.060	2.160	1311.2
**DIANA**	0.361	0.065	2.336	1022.2
**Fuzzy c-means**	0.154	0.113	1.905	682.6
**K-means**	0.179	0.087	1.964	897.7
