# Supplementary material for: Application of ensemble clustering and survival tree analysis for identifying prognostic clinicogenomic features in patients with colorectal cancer from the 100,000 Genomes Project
Source: BMC Res Notes. 2021 Oct 2;14:385. doi: 10.1186/s13104-021-05789-0 (PMC8487486; doi:10.1186/s13104-021-05789-0)
Supplement: Supplementary file 4 — Additional file 4: Internal cluster validity indices for k = 4. [file 13104_2021_5789_MOESM4_ESM.pdf]

Additional file 4: Internal cluster validity indices for k = 4

Algorithm	C-index	Silhouette	Compactness	Connectivity
**PAM**	0.301	0.077	2.239	860.7
**DIANA**	0.368	0.045	2.360	1048.8
**Fuzzy c-means**	0.140	0.165	1.988	467.5
**K-means**	0.150	0.151	2.006	607.7
**Ensemble**	0.139	0.168	1.991	427.7
