# Supplementary material for: Application of ensemble clustering and survival tree analysis for identifying prognostic clinicogenomic features in patients with colorectal cancer from the 100,000 Genomes Project
Source: BMC Res Notes. 2021 Oct 2;14:385. doi: 10.1186/s13104-021-05789-0 (PMC8487486; doi:10.1186/s13104-021-05789-0)
Supplement: Supplementary file 5 — Additional file 5: Clinicogenomic features after ensemble clustering. [file 13104_2021_5789_MOESM5_ESM.pdf]

Additional file 5: Clinicogenomic features after ensemble clustering

	Cluster 1 (n = 1001)		Cluster 2 (n = 702)		Cluster 3 (n = 230)		Cluster 4 (n = 278)	
**Male (%)**	581	58.0%	484	68.9%	116	50.4%	138	49.6%
**Age groups (%)**								
≤ 30	3	0.3%	1	0.1%	0	0.0%	0	0.0%
31-50	79	7.9%	83	11.8%	0	0.0%	15	5.4%
51-70	383	38.3%	487	69.4%	9	3.9%	161	57.9%
≥ 71	536	53.5%	131	18.7%	221	96.1%	102	36.7%
**Tumour stage (%)**								
1	193	19.3%	71	10.1%	0	0.0%	97	34.9%
2	499	49.9%	155	22.1%	20	8.7%	128	46.0%
3	245	24.5%	402	57.3%	181	78.7%	34	12.2%
4	64	6.4%	74	10.5%	29	12.6%	19	6.8%
**Tumour grade (%)**								
1	28	2.8%	26	3.7%	6	2.6%	7	2.5%
2	786	78.5%	606	86.3%	173	75.2%	245	88.1%
3	187	18.7%	70	10.0%	51	22.2%	26	9.4%
**Treatment (%)**								
**Chemotherapy**	296	29.6%	528	75.2%	146	63.5%	77	27.7%
**Radiotherapy**	38	3.8%	78	11.1%	29	12.6%	24	8.6%
**Immunotherapy**	10	1.0%	10	1.4%	5	2.2%	5	1.8%
**Tumour mutational burden (mean, SD)**	32.3	±47.8	4.4	±7.3	11.9	±22.2	5.27	±7.8
***KRAS* mutation (%)**	144	14.4%	335	47.7%	114	49.6%	169	60.8%
***BRAF* mutation (%)**	169	16.9%	273	38.9%	97	42.2%	155	55.8%
***NRAS* mutation (%)**	95	9.5%	229	32.6%	73	31.7%	152	54.7%
**1-year survival**	95.7%		98.7%		87.8%		96.8%	
**2-year survival**	90.4%		96.7%		70.0%		90.6%	
**3-year survival**	86.5%		93.0%		53.1%		79.9%	
